# Measurement of polarization effects in dual-phase ceria-based oxygen permeation membranes using Kelvin probe force microscopy

**DOI:** 10.3762/bjnano.12.102

**Published:** 2021-12-15

**Authors:** Kerstin Neuhaus, Christina Schmidt, Liudmila Fischer, Wilhelm Albert Meulenberg, Ke Ran, Joachim Mayer, Stefan Baumann

**Affiliations:** 1 Forschungszentrum Jülich GmbH, Institute of Energy and Climate Research 12, Helmholtz-Institute Münster: Ionics in Energy Storage, Corrensstr. 46, 48149 Münster, Germany; 2 Forschungszentrum Jülich GmbH, Institute of Energy and Climate Research, Materials Synthesis and Processing (IEK-1), Wilhelm-Johnen-Straße, 52452 Jülich, Germany; 3 Faculty of Science and Technology, Inorganic Membranes, University of Twente, 7500 AE Enschede, Netherlands; 4 Central Facility for Electron Microscopy GFE, RWTH Aachen University, 52074 Aachen, Germany; 5 Ernst Ruska-Centre for Microscopy and Spectroscopy with Electrons ER-C, Forschungszentrum Jülich GmbH, 52425 Jülich, Germany

**Keywords:** ceria, diffusion coefficient, Kelvin probe force microscopy, oxygen permeation

## Abstract

In this study, a dual phase composite (CSO-FC2O) consisting of 60 vol % Ce_0.8_Sm_0.2_O_1.9_ as oxygen-conductive phase and 40 vol % FeCo_2_O_4_ as electron-conductive phase was synthesized. TEM measurements showed a relatively pure dual-phase material with only minor amounts of a tertiary (Sm,Ce)(Fe,Co)O_3_ perovskite phase and isolated residues of a rock salt phase at the grain boundaries. The obtained material was used as a model to demonstrate that a combination of polarization relaxation measurements and Kelvin probe force microscopy (KPFM)-based mapping of the Volta potential before and after the end of polarization can be used to determine the chemical diffusion coefficient of the ceria component of the composite. The KPFM measurements were performed at room temperature and show diffusion coefficients in the range of 3 × 10^−13^ cm^2^·s^−1^, which is comparable to values measured for single-phase Gd-doped ceria thin films using the same method.

## Introduction

Acceptor-doped cerium dioxide, where cerium is partially substituted by cations of lower valence (most prominently Gd^3+^), is a fluorite material with a very high oxide ion conductivity at comparably moderate temperatures (around 600 °C). It has already been in focus of research for roughly 50 years [1]. The ion conductivity is combined with a moderate electron conductivity, which strongly depends on the oxygen partial pressure [2–4]. These features make ceria an interesting material for high-temperature industrial applications, for example, as oxygen permeation membrane, as oxygen sensor material, or for the use in solid oxide fuel cell components [1,5–6]. Apart from this, ceria is also widely employed as a catalyst in the middle- to low-temperature regime (20–400 °C) [7–9], making ceria-based dual-phase materials with a second electron-conductive spinel or perovskite phase applicable in membrane reactors for partial oxidation reactions.

Dual-phase membranes with FeCo_2_O_4_, or its iron-rich pendant Fe_2_CoO_4_, and Gd-doped ceria as an ion conductor have already been successfully applied as oxygen permeation membranes with high permeability in the temperature range above 800 °C [10–12], and microstructural as well as mechanical investigations and optimizations have been performed [13–15]. Especially for FeCo_2_O_4_ addition, the development of an additional electron-conductive phase (namely (Ce,Gd)(Fe,Co)O_3_), whose composition and abundance depends on the ceria/spinel ratio, has recently been discussed [16]. Current research efforts are aiming to improve the composition and microstructure of a dual-phase membrane with similar composition for application in a membrane reactor at considerably lower temperatures (below 600 °C) to perform partial oxidation reactions. Apart from Gd-doped ceria, Sm-doped ceria also could be an interesting alternative in this kind of composite due to its high ionic conductivity [17].

Obstacles that generally still need to be addressed for the application of dual-phase ceria-based membranes in such an environment are the strong decrease in oxygen ion mobility below 600 °C, mechanical stability and chemical stability against the used reactive gases, and local chemical reactions at the triple-phase boundaries (ceria|electron conductor|gas) as well as at the interfaces between the different components (ceria|ceria, ceria|electron conductor, and electron conductor|electron conductor).

Kelvin probe force microscopy (KPFM) is an atomic force microscopy (AFM)-based measurement method that can measure the local surface potential (or Volta potential) of the sample [18–19]. The surface potential is a sensitive indicator for local changes of the defect chemistry, as it is directly related to the local Fermi level [20]. The defect chemistry of acceptor-doped ceria and the oxide ion/electronic transport within ceria single-phase materials and also for ceria-based dual-phase materials is well understood at temperatures above 400 °C. However, the implications of local oxidation and reduction for charge carrier mobility at lower temperatures are by far less well studied. Therefore, a combined polarization-KPFM experiment was performed on a dual-phase material consisting of 60 vol % Ce_0.8_Sm_0.2_O_1.9_ and 40 vol % FeCo_2_O_4_ (CSO-FC2O) as electron-conductive phase in order to, first, locally change the defect chemistry of the material and, then, study the relaxation to the original surface potential state during uptake/release of oxygen from/to the surrounding air.

By using an AFM tip as an electron-conductive nanoscale electrode, a constant voltage pulse was applied to the sample in order to achieve a local polarization with distinctly changed redox state and defect concentrations. In a subsequent mapping experiment, the AFM tip was used as Kelvin probe to scan the locally changed surface potential distribution at the sample surface until the original state was reached again. In this way, polarization-induced, reversible surface potential changes have already been monitored for a variety of single-phase acceptor-doped ceria materials [21–23]. Time-resolved relaxation curves calculated from the KPFM images can be used to determine room temperature diffusion coefficients [24].

These data are not easily accessible by oxygen permeation experiments or state-of-the-art electrochemical experiments with macroscopic electrodes, because the electrical conductivity of ceria-based materials is very low below 400 °C. An additional obstacle for measuring the response of the ion-conductive material in a composite including an additional electron-conductive phase is that the electron conductivity normally is so high that it usually obscures the contribution from the ion conductor. Therefore, measurements with small contacts are needed, where the electron and ion conducting phase can be addressed separately, making AFM-based electrochemical measurements predestined for detailed analyses of the constituents of composite materials.

## Theoretical Background

### Kelvin probe force microscopy

The presented measurements were performed in a single-pass experiment. For this kind of measurements, the surface potential and the sample topography are mapped in a single pass in intermittent contact mode with the cantilever vibrating at its resonance frequency (i.e., the cantilever is not in lift mode during this experiment as it would be for the more common dual-pass experiment).

To yield a surface potential measurement, an AC potential with a certain frequency and amplitude is applied to the AFM tip. This AC potential is fixed by an additional external voltage *U*_DC_. The external voltage compensates the surface potential difference between sample and probe and is used for the KPFM map. The connection between the KPFM signal *U*_KPFM_ and the applied voltage is illustrated in the following equation:


[1]

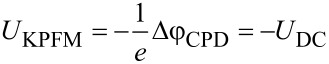



### Effect of polarization

In previous experiments it was already observed, that polarizing single-phase, acceptor-doped ceria materials led to a more positive surface potential in the direct vicinity of the contact area, while a negative voltage led to a more negative surface potential. The surface potential gradient was also shown to be reversible over time as long as the applied voltage was kept in a certain range. The time dependence was shown to vary with dopant concentration (e.g., abundance of oxygen vacancies in ceria) and also depends on the ratio of grain boundary/grain bulk [22–25]. Single ceria grains in a mixed ion/electron-conductive composite have so far not been addressed by AFM-based electrochemical studies.

The surface potential at the direct contact point of the measuring tip can be determined from the KPFM measurement data at different times after the end of polarization. The results usually follow an exponential rule if plotting ΔΦ_SP_ versus time. By fitting of the expontential function, the time constant of the relaxation curve (τ_fit_) can be calculated. τ_fit_ = 
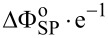
 which is 
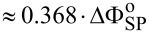
 with 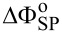 being the surface potential difference at the end of the polarization/beginning of the relaxation process. An initial strong decrease/increase of the potential instantaneously after the end of the polarization is to be expected, because of the still measurable electron conductivity of the spinel at room temperature and the possibility of redistribution of electrons over the uppermost layers of the surface of the ceria phase [26–28].

For macroscopic polarization experiments, τ_fit_ can be expressed by multiplying the chemical capacitance *C*^δ^ and chemical resistance *R*^δ^ [24,29]:


[2]

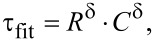



where


[3]

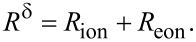



Here *R*_ion_ is the ionic contribution to resistance and *R*_eon_ is the respective electronic contribution.

The calculated time constant τ_fit_ (in s) can subsequently be used (in the case of macroscopic as well as AFM-based polarization experiments) to determine the chemical diffusion coefficient *D*^δ^ (in cm·s^−1^) by applying


[4]

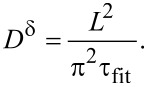



In ceria, the chemical diffusion coefficient is thought to be the diffusion coefficient for oxygen ions. The diffusion length *L* (in cm) is, in the case of the AFM-based measurements, the length scale that can be obtained from the lateral dimension (e.g., distance from the contact area to rim of affected region) of the surface potential gradient introduced by the polarization experiment. Depending on the voltage applied during our experiments, it ranged roughly between 200 and 300 nm. This estimate is one of the largest sources of error in the calculation for dual-phase materials as, in contrast to the defect gradient observed for single-phase materials where the gradient was more or less circular around the contact area, the shape of the introduced defect gradient in the dual-phase membranes observed in this study strongly depended on the surrounding.

In single-phase ceria materials, oxygen is incorporated into oxygen vacancies in the structure at high temperatures [4,30]:


[5]

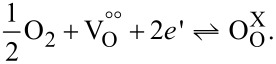



Previous studies showed that at temperatures below 400 °C the concentration of defect associates in ceria increases because electrons are trapped at oxygen vacancy sites, allowing also for singly charged (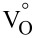) or uncharged (V_O_) oxygen vacancies in close vicinity to Ce^3+^ ions [31–32], which show a strongly lowered mobility. For low temperatures, the common electroneutrality equation for acceptor-doped ceria can be shortened, as oxygen on interstitial lattice sites (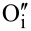 and 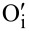) is negligible at or below atmospheric oxygen partial pressure:


[6]

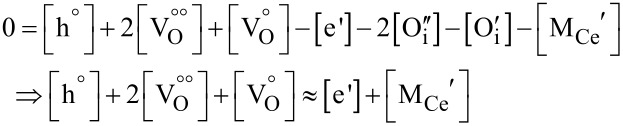



In the case of the composite material in this study, the electron conductor FeCo_2_O_4_ has to be taken additionally into account to anticipate the reaction to local polarization. Generally, an electron conductor should lead to a very fast relaxation after a polarization, at least for measurements with macroscopic contacts. Fe and Co are both redox-active cations, though, and can be reduced or oxidized under relatively mild conditions. Therefore, it is possible, that applied potentials above a certain threshold voltage can lead either to oxidation or reduction of the Fe, Co, or even Ce in the samples (depending on the sign of the applied potential). This would lead to an increase or decrease in the slope of the polarization curve. If the reaction is reversible, it would depend on the velocity of the backreaction whether it would be visible in the relaxation curve. If the backward reaction was slower than the general relaxation of the material, the relaxation would not follow an exponential rule anymore and could also show an additional plateau or “bump”.

## Experimental

### Sample preparation

Commercial powders of Ce_0.8_Sm_0.2_O_2−δ_ (CSO, Kceracell, Korea), Fe_2_O_3_ and Co_3_O_4_ (Sigma-Aldrich, Germany) were used for solid-state reactive sintering. Respective amounts of powders were weighed for nominal CSO-FeCo_2_O_4_ compositions with a weight percent ratio of 60:40. The powder mixture was ball milled in ethanol for 48 h on a roller bench with 175 rpm. After drying in ambient air at 70 °C the powder mixture was pressed with an uniaxial press in disc-shaped membranes with *d* = 20 mm. The discs were sintered with a heating rate of 5 K/min to 1200 °C and a dwell time of 5 h. At the sintering temperature, the spinel is partially reduced into a high-temperature monoxide phase with rock salt structure. Therefore, a slow rate of 0.5 K/min between 900 and 800 °C is implemented in the cooling cycle in order to enable complete re-oxidation of the high-temperature Co/Fe monoxide to the respective spinel phase according to the Fe_3−_*_x_*Co*_x_*O_4_ phase diagram [10].

For electrical conductivity measurements, the samples were burnished using sanding paper (1200 graining). For KFPM measurements, the samples were embedded in epoxy resin and polished to mirror using diamond polishing paste. The roughness of the polished samples was around 50 nm.

### Electrical conductivity measurements

The electrical conductivity was determined in a DC measurement where the sample was put between two Pt contacts, one made of a Pt wire with a contact diameter of 700 µm and one made of a Pt sheet with additional Pt resinate paste (RP 070107, Heraeus GmbH, Germany) for improved contact, and polarized with either +200 mV or −200 mV for 300 s referring to the microscale contact. Subsequently, the relaxation was observed by measurement of the open-circuit potential for 600 s. Measurements were performed using a 2400 sourcemeter (Keithley, USA) in ambient air and in a temperature range between 100 and 800 °C. Below 100 °C, especially the relaxation measurements became too noisy for evaluation.

### KPFM measurements

AFM measurements were performed applying KPFM-AFM mode in ambient air at room temperature using a 5500 AFM by Keysight Technolgies (USA). Cantilevers with a conductive coating or a Pt/Ir alloy (PPP-NCSTPt by Nanosensors, Switzerland) were used for the KPFM measurements. In the present study, grains with a high surface potential were chosen for polarization experiments, as they are likely to represent ceria, while electron-conductive phases should show a comparably low surface potential in the unpolarized state. This is because the Fermi level of the ceria sample is lower than that of the spinel resulting in a larger potential difference between the ceria and the Pt/Ir coating of the tip than for the spinel. After the end of polarization, KPFM measurements were started with an imaging velocity of 1 image per minute to measure the relaxation of the introduced gradient.

### Electron microscopy

The TEM specimens were cut from 60CSO20-FC2O pellets by focused ion beam (FIB) milling using a FEI Strata400 system with Ga ion beam. Further thinning and cleaning were performed with an Ar ion beam in a Fischione Nanomill 1040 at 900 eV and 500 eV beam energy, respectively. TEM and energy-filtered TEM (EFTEM) imaging were performed with a FEI Tecnai F20 at 200 kV. High-resolution HAADF imaging and energy-dispersive X-ray (EDX) chemical mapping were conducted with a FEI Titan G2 80-200 ChemiSTEM microscope equipped with an XFEG, a probe Cs corrector and a super-X EDXS system.

## Results and Discussion

### Electron microscopy

**Figure 1:**
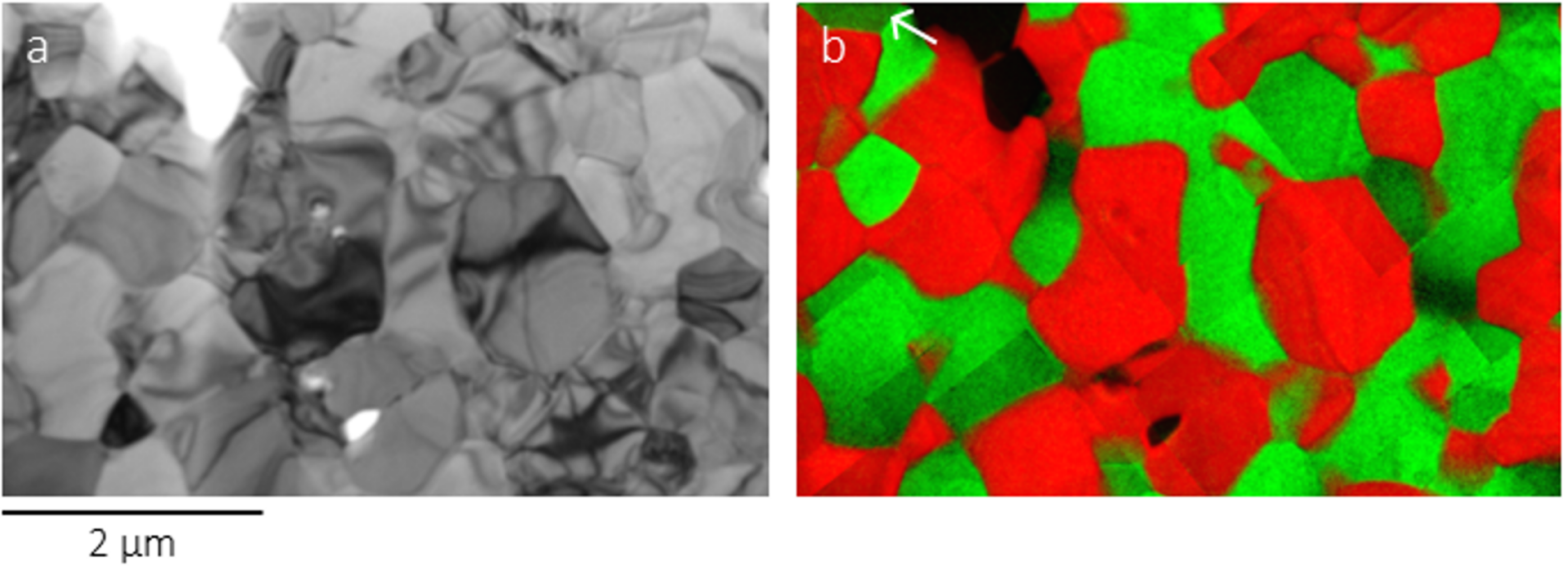
TEM image (a) and the corresponding energy-filtered TEM (EFTEM) image (b) showing the grain size and phase distribution inside 60CSO20-FC2O (red: Ce and thus representing CSO, green: Co and thus representing FC2O). The arrow in (b) indicates a SmFeO_3_ grain.

A representative grain and phase distribution image of the sample is shown in Figure 1. The EFTEM image in Figure 1b clearly shows two separate phases, CSO in red and FC2O in green, revealing a homogeneous mixture of the two phases. A few grains of the tertiary phase SmFeO_3_ (SFO) with space group *Pbnm* and Ce/Co partially taking the Sm/Fe sites can be occasionally observed. In the case of Figure 1, only a single SFO grain is noticed, as indicated by the arrow in the upper left side in Figure 1b.

**Figure 2:**
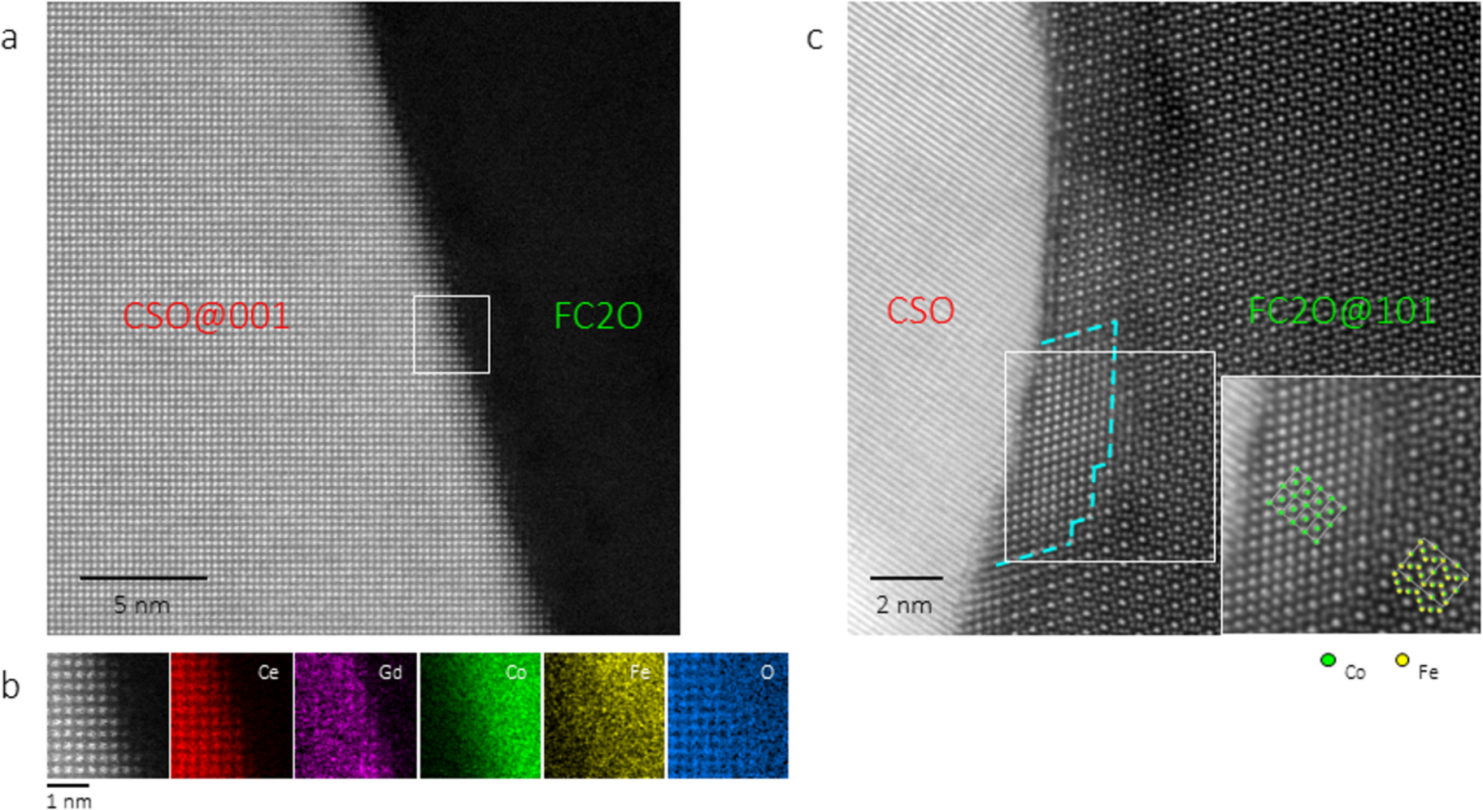
Interfaces between CSO and FC2O. (a) HAADF image of a CSO-FC2O interface, with CSO oriented along the [001] direction and FC2O randomly oriented. (b) EDX chemical mapping from the square-defined-region in (a): HAADF image and the elemental maps from Ce L line, Sm L line, Co K line, Fe K line, and O K line. (c) HAADF image of a CSO-FC2O interface, with FC2O oriented along the [101] direction and CSO randomly oriented. The dashed line outlines a rock salt structure on the FC2O side of the interface. The square-defined region is enlarged in the lower-right inset, with correspondingly oriented structural models. Oxygen is not shown.

The HAADF image in Figure 2a shows a CSO-FC2O interface with the left CSO grain tilted along its [001] direction. A clean surface without any significant structural defects is observed. Corresponding to the square-defined region in Figure 1a, Figure 2b displays the EDX chemical mapping results with atomic resolution, indicating no chemical disorders except the slight enrichment of Sm at the edge of the CSO grain. Tilting FC2O along its [101] direction, Figure 2c shows another CSO-FC2O interface, where rock salt structures within several nanometers are located on the FC2O side as outlined by the dashed line. The enlarged interface structure in the lower-right inset shows a good agreement with the CoO and Fe_3−_*_x_*Co*_x_*O_4_ structural model, respectively.

### Electrical conductivity

**Figure 3:**
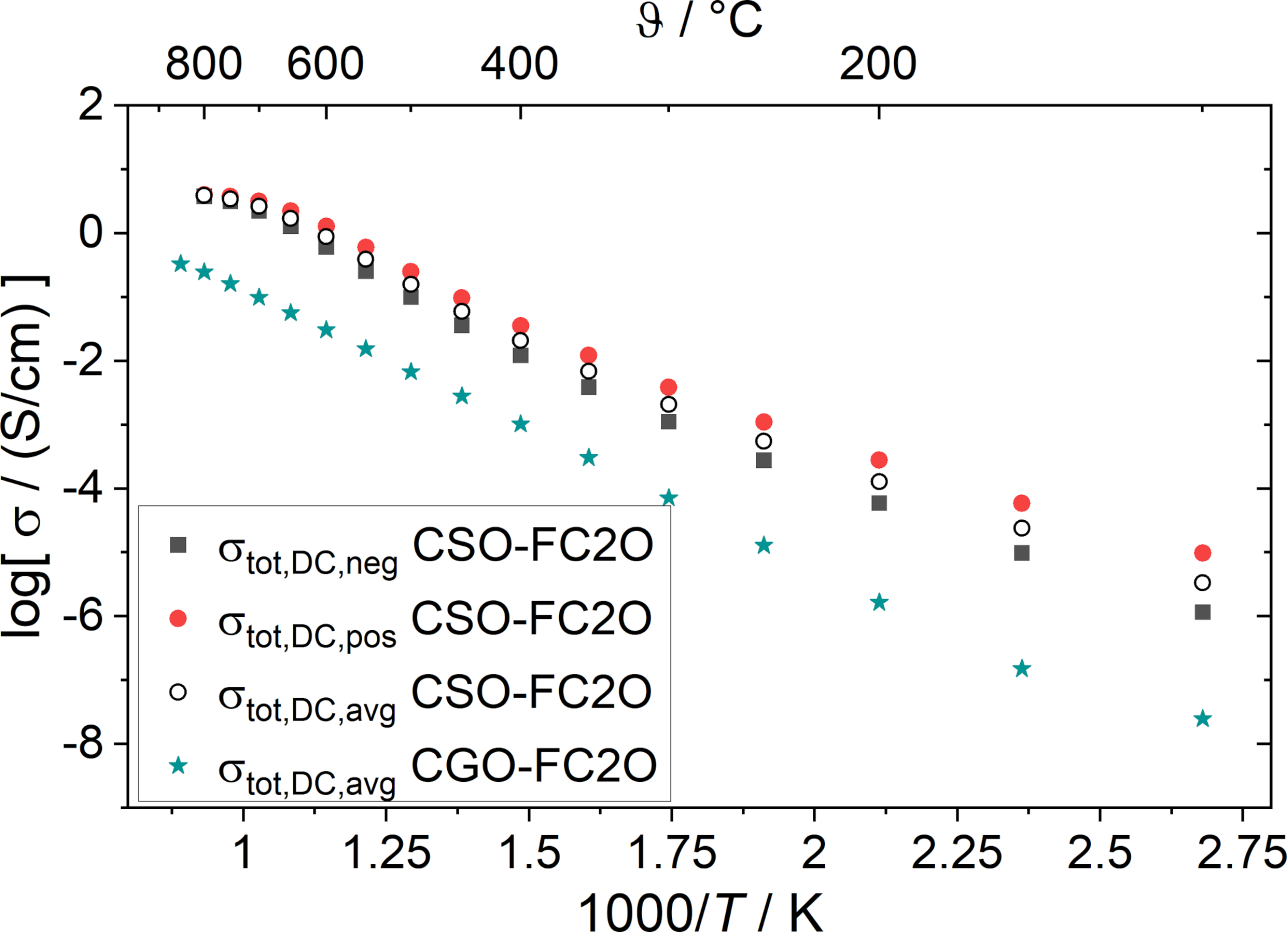
Electrical conductivity obtained in air by application of either +200 mV or −200 mV and analyzing the steady-state current at temperatures between 100 and 800 °C. Activation energies calculated from the average conductivity are 65 ± 1 kJ·mol^−1^ at 100–400 °C and 91 ± 3 kJ·mol^−1^ between 400 and 700 °C with a plateau above 700 °C. The green stars show the average total conductivity of 60 wt % Ce_0.8_Gd_0.2_O_1.9_ and 40 wt % FeCo_2_O_4_ produced by solid-state sintering measured with the same measurement method as in [16].

The sample composition with Sm instead of Gd used for this study shows a clearly increased conductivity compared to the well-known 60 vol % Ce_0.8_Gd_0.2_O_1.9_ and 40 vol % FeCo_2_O_4_ (CGO-FC2O) over the whole measured temperature range (Figure 3).

Measurements of the electrical conductivity, in addition, showed a difference between the application of a positive or a negative voltage, which was especially strong at low temperatures (Figure 3). The steady state during polarization was achieved more or less instantly for all temperatures. The polarization was performed for 5 min and then switched off. The relaxation of the sample was subsequently investigated by measuring the open-circuit potential. For all temperatures, the relaxation took less than 40 s for negative polarization and less than 60 s for positive polarization. All relaxation curves showed a clear dependence on exponential rules, although for temperature below 250 °C, the signal-to-noise ratio was very low for the relaxation measurements. Measurements for temperatures below 100 °C were not analyzable.

### Positive polarization and KPFM

Similar to KPFM-based polarization–relaxation analyses performed for single-phase ceria materials [21–25], positive polarization of CSO grains in the CSO-FC2O composite with a tip bias of +3 V led to a locally increased surface potential reaction, which decreased over time.

**Figure 4:**
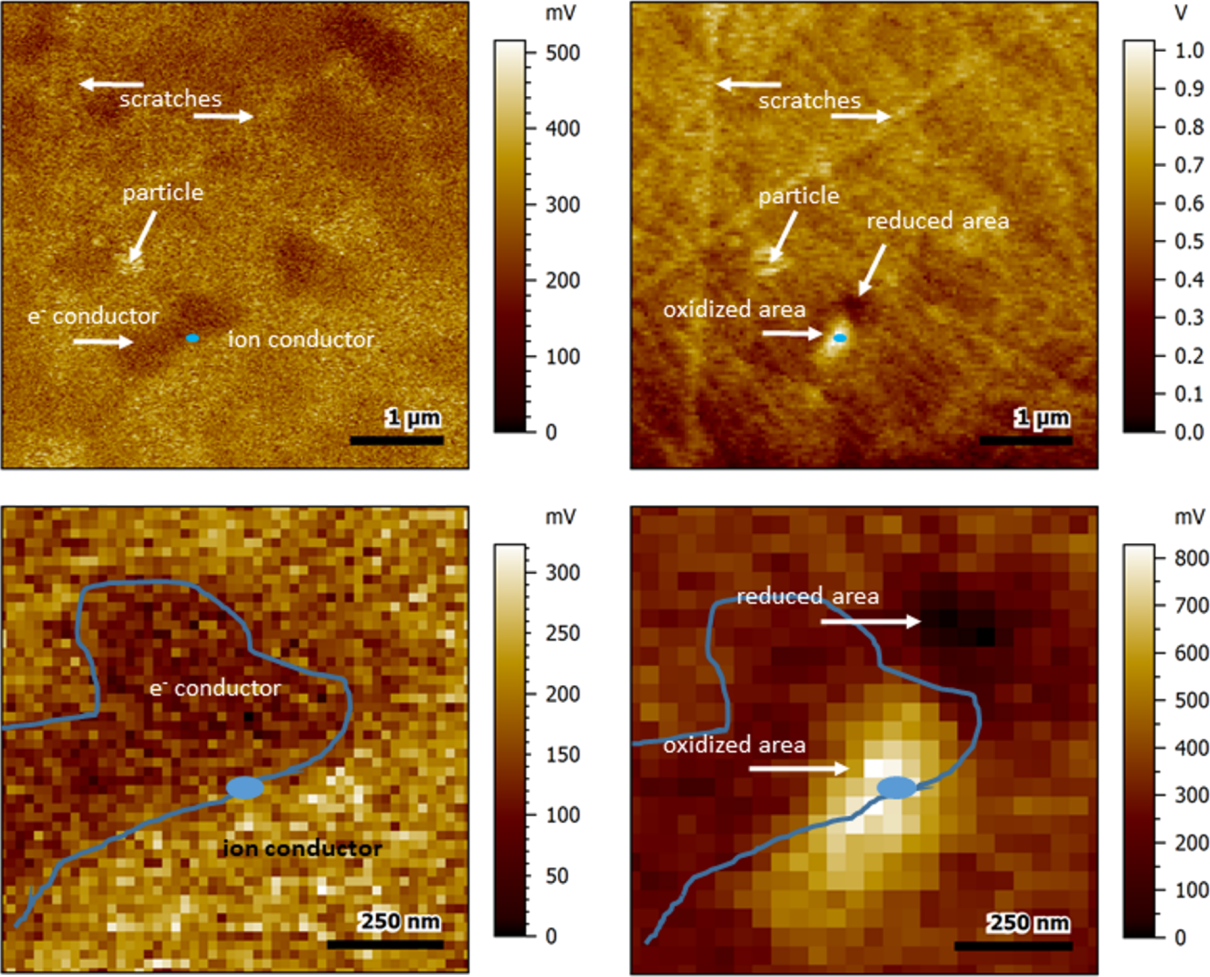
Surface potential images of the sample before (left) and after (right) polarization with +1 V for 60 s. The images at the bottom show a zoomed-in version of the contact area. The contact area is indicated by the blue dot. The blue line shows the estimated grain boundary between electron-conductive and ion-conductive phase.

For a tip bias of +1 V, in some cases, there was not only an area with an increased surface potential in direct vicinity to the contact area, but an area with a reduced surface potential close to this region was also observable. The parallel reactions occurred when the polarization was performed at the edge between a region with higher surface potential (indicating the ceria ion conductor) and a region with lower surface potential (indicating the electron-conductive material) as shown in Figure 4. Here, the contact area for polarization, shown as blue dot, was deliberately placed at the interface between a grain with high surface potential (assigned to the ceria component) and a grain with lower surface potential (assigned to the electron conductor).

The region with reduced surface potential after polarization was found at the location of the ceria material, while the local increase of surface potential was found to affect both the ceria grain and the adjacent electron conductor. For both, a time-dependent relaxation process was observed, although the relaxation process of the region with increased surface potential did not follow an exponential rule. It was possible to calculate a time constant from the region with the reduced surface potential, though (see also Figure 7 and Table 1 in the Discussion section).

### Negative polarization and KPFM

In contrast to positive polarization, polarizing the sample with a negative bias referring to the AFM tip led to a lowered surface potential in the direct vicinity of the contact, which is in good accordance with the results previously published for single-phase ceria materials [21–25].

**Figure 5:**
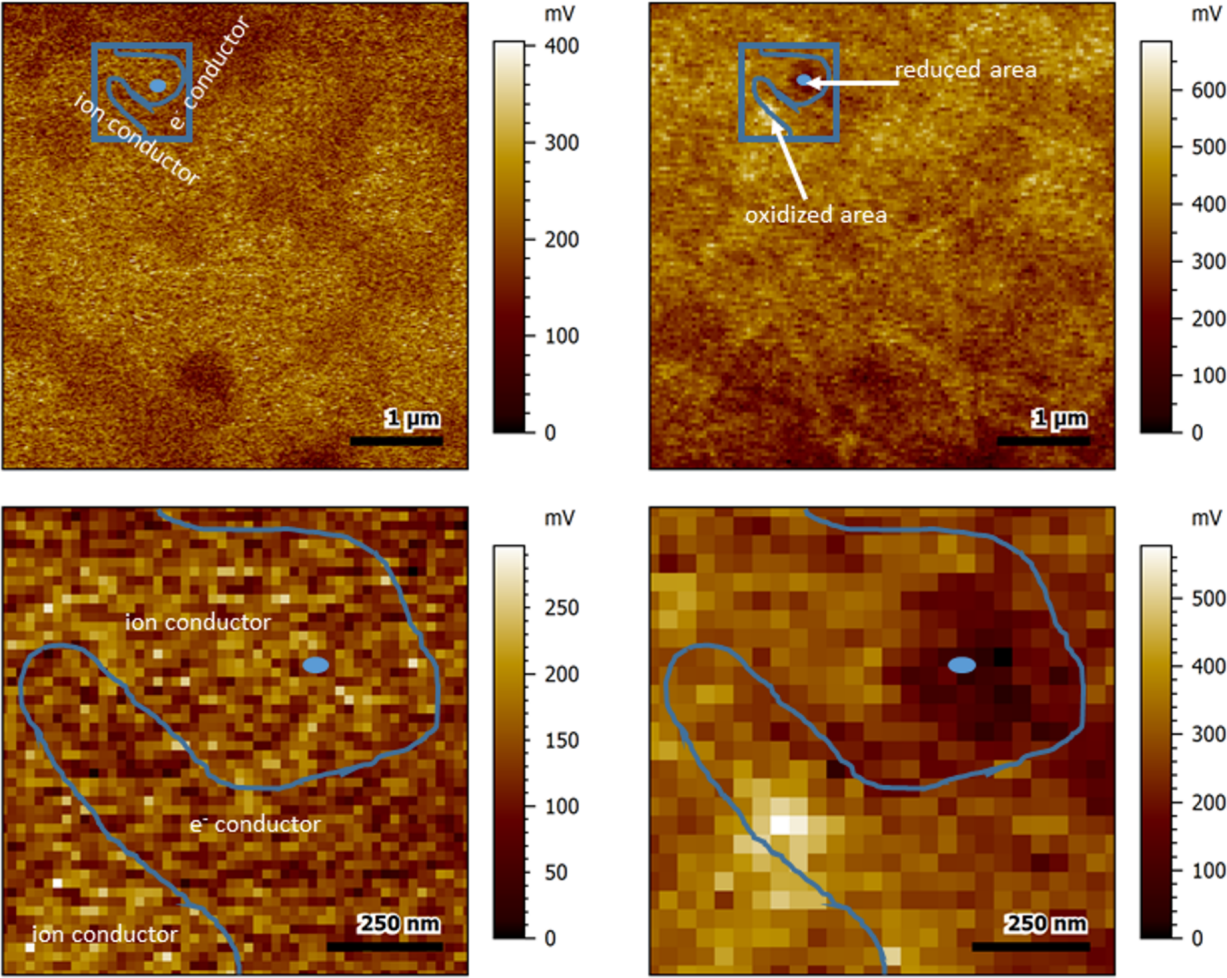
Surface potential images of the sample before (left) and after (right) polarization with −1 V for 60 s. The images at the bottom show a zoomed-in version of the contact area. The contact area is indicated by the blue dot. Blue lines show a rough sketch of the estimated grain boundary between ion-conductive phase and electron-conductive phase. The blue square in the top-most images shows zoomed-in region.

Similar to the effect observed with +1 V polarization, polarization with a tip bias of −1 V led in some cases to a parallel oxidation effect of an area adjacent to the contact area (Figure 5). This secondary process did not show an exponential relaxation behavior (see also Figure 7 in the Discussion section). Hence, it was not possible to calculate a time constant for this process.

Since the opposite reaction with a second potential gradient observed after polarization with +1 V as well as −1 V was found only when the polarization occurred directly at or on a grain boundary between ceria and electron-conducting material, one possible explanation is that the spinel also participates in the reaction in this case. Speculatively, it would be possible that the spinel, whose iron and cobalt moieties are also redox active, could lead to further charge redistribution and additional reaction. In this case, however, the additional gradient would be expected to be in the spinel region rather than on another site of the ceria phase. Another possible explanation would be that enhanced charge transport by electrons occurs along the grain boundary. This has already been confirmed experimentally for similar composite materials [33].

In this case, an opposite reaction could appear at a different point or along the entire length of the grain boundary. Since the “opposite reactions” we found in the cases observed so far always occurred at locations along the grain boundary, this is more likely. However, it is not clear why the opposite reaction does not occur along the entire length of the grain boundary and is instead localized in one area.

Neither for the positive nor for the negative polarization experiments, a change of the local sample topography was observed, which would hint at a local phase transformation. As the surface potential also relaxes to the original state (although this took longer for polarization with ±3 V, where the original state was only reached after more than 1 h), it can be safely assumed that there is no irreversible reaction taking place during polarization.

## Discussion

TEM measurements showed that the sample consists mainly of Ce_0.8_Sm_0.2_O_1.9_ and FeCo_2_O_4_ with only minor amounts of the perovskite phase (Sm,Ce)(Fe,Co)O_3_. SFO is thought to be a mainly electron-conductive material, as the related phase (Gd,Ce)(Fe,Co)O_3_, which develops in composites of Ce_0.8_Gd_0.2_O_1.9_ and FeCo_2_O_4_ is an electron conductor as well [16]. The interfaces between CSO and FC2O have been shown to be mainly clean with only a slight segregation of Sm. Additionally, in some areas residues of a rock salt phase have been found. This phase is prone to develop during the synthesis process of CSO-FC2O depending on the sintering parameters. The formation can be more or less completely suppressed by slow cooling during the sintering process, as the rock salt phase mainly forms in a certain window of temperature and at increased Fe concentrations of the spinel [10]. On the whole, it can be assumed that no interconnected grain boundary phases or strongly elevated concentrations of electron-conducting elements have accumulated on the grain boundaries between CSO and FC2O, which could possibly influence the effect of charge distribution during or after polarization with the AFM tip in direct vicinity of CSO-FC2O grain boundaries.

However, independent from the sign of the polarization, it was observed in some cases that charge is obviously distributed along scratches on the surface, which were introduced by polishing. This underlines the hypothesis, that fast charge transport in ceria-based materials via the uppermost surface layers is still possible at very low temperatures, while the charge transport in the bulk of the material is strongly reduced compared to “usual” operation temperatures (600–900 °C), due to freezing of the oxygen vacancies and formation of defect accumulates [32].

**Figure 6:**
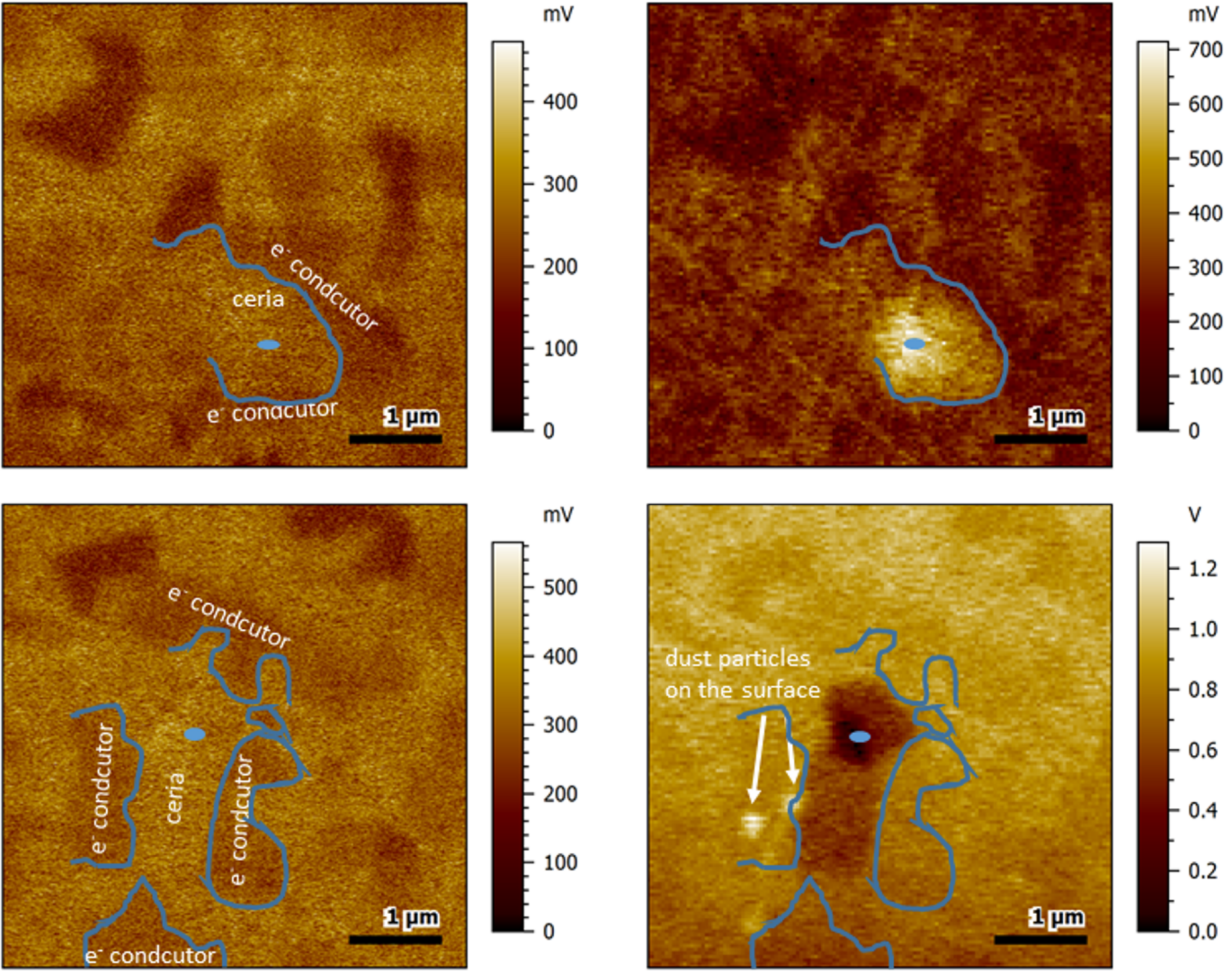
Surface potential images before (left) and directly after polarization (right) with either +3 V (top row) or −3 V (bottom row). The blue dot shows the area where the AFM tip was placed during polarization. The rough sketches illustrate the grain boundaries at the interface between the polarized ceria grain and the surrounding electron-conductive grains.

At the same time, the direct polarization effect was confined to grains of the same composition and did not spread over phase boundaries. An example where this can be very well observed is in Figure 6. There is an intensely affected area after polarization with −3 V for 60 s with a diameter of roughly 500–600 nm, but a less strong effect also spreads out to an area above and below the contact area. The region with lowered surface potential is effectively confined by the surrounding grains of electron-conductive material.

When comparing DC-2-point and AFM-based polarization–relaxation experiments, the relaxation process observed with macroscopic contacts was much faster than the relaxation process observed via KPFM, where (depending on tip bias) the relaxation process in most cases took more than 30 min. For the polarization with the AFM tip, higher voltages and a much smaller contact diameter were used, though, resulting in a significantly increased current density at the tip–sample contact compared to the Pt microscale contact in the DC measurement setup. In addition, when measuring DC-2-point measurements with macroscopic contacts, the spinel and the ceria part of the composite are both probed, so that a charge redistribution via the electron-conductive spinel phase will always occur. This is a much faster process than charge redistribution in the ceria material, which was specifically probed by the AFM-based experiments, leading to the observation of a longer relaxation process in case of the AFM-based measurements.

**Figure 7:**
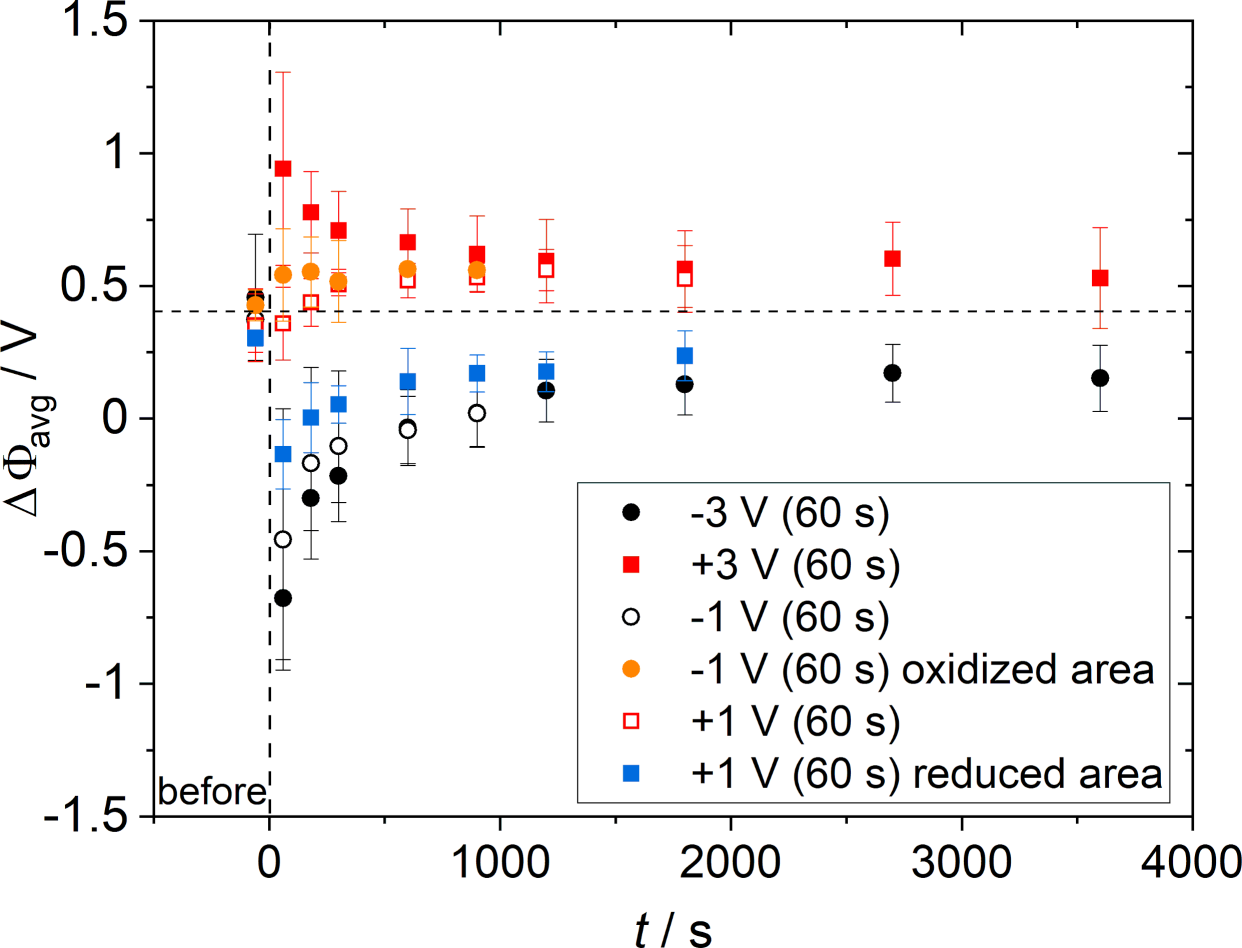
Comparison of the average relaxation curves of the surface potential after polarization. For polarization with +1 V or −1 V, in some cases a reduced or oxidized area was found in close vicinity to the oxidized or reduced contact area, which is assumed to be the result of a secondary reaction in the material. The respective relaxation of the secondary reaction is shown in orange (area with increased surface potential after polarization with −1 V) or blue (area with reduced surface potential after polarization with +1 V). The dotted line is the average surface potential at the contact position before polarization.

In Table 1, the calculated time constants as well as the chemical diffusion coefficients and the diffusion length *L* used for the calculation from the average relaxation curves in Figure 7 are enlisted. For positive polarization with either +1 or +3 V, *D*^δ^ is slightly higher compared to negative polarization. This is in good accordance with the conductivity measurements, where the conductivity was slightly lower and the activation energy slightly higher for experiments with negative polarization. The values obtained for the Sm-doped ceria in the composite in this study show also good agreement to data obtained for Gd-doped ceria thin films using the same experimental setup [24].

**Table 1:** Calculated time constants and diffusion coefficients at room temperature in comparison to literature values for single-phase Ce_0.9_Gd_0.1_O_1.95_ thin films from [24] obtained with the same method. The diffusion length *L* was determined from the average radius of the area affected by polarization, which could be measured in the KPFM map. The error of the chemical diffusion coefficient is in the range of 10% because the diffusion length was in several cases variable due to an irregular shape of the potential gradient.

Material	Experiment	τ_fit_ [s]	*L* [nm]	*D*^δ^ [cm^2^·s^−1^]

Ce_0.9_Gd_0.1_O_1.95_ epitactic thin film [24]	−5 V for 300 s	132	200	3.07 × 10^−13^
Ce_0.9_Gd_0.1_O_1.95_ nanocrystalline [24]	+5 V for 300 s	186	200	2.18 × 10^−13^
Ce_0.9_Gd_0.1_O_1.95_ epitactic thin film [24]	−3 V for 300 s	310	200	1.27 × 10^−13^
Ce_0.9_Gd_0.1_O_1.95_ epitactic thin film [24]	+3 V for 300 s	479	200	8.74 × 10^−14^
CSO-FC2O	−3 V for 60 s	274	300	3 × 10^−13^
CSO-FC2O	+3 V for 60 s	202	300	5 × 10^−13^
CSO-FC2O	−1 V for 60 s	200	200	2 × 10^−13^
CSO-FC2O (reduced area)	+1 V for 60 s	116	200	4 × 10^−13^

As the introduced gradient in the composite material was in most cases not circular as observed for the single-phase materials, one of the biggest sources of error for the calculation of the diffusion coefficients shown in Table 1 is the determination of the diffusion length *L*. This is the reason, why the number of significant digits is reduced for the present measurements compared to the measurements from [24].

## Conclusion

It has been shown that using an AFM tip for addressing single grains in a composite material and analyzing their polarization–relaxation behavior by means of Kelvin probe force microscopy is a way to work out diffusion coefficients of respective constituents in a composite material.

The next step concerning the AFM-based analysis of composite materials is the combination of local chemical analysis methods (e.g., EDX or EELS) with local electrochemical experiments or KPFM imaging. Additionally, temperature-dependent measurements of the polarization–relaxation behavior are planned.
